# Cohort profile: Norwegian survey of health and ageing (NORSE)

**DOI:** 10.1186/s12889-021-12294-3

**Published:** 2021-12-08

**Authors:** Bjørn Heine Strand, Vegard Skirbekk, Ellen Melbye Langballe, Sverre Bergh, Brynjar Landmark, Sigrid Wangensteen, Geir Selbæk, Øyvind Kirkevold

**Affiliations:** 1 Norwegian Institute of Public Health, P.O. Box 222 Skøyen, 0213 Oslo, Norway; 2 Norwegian National Advisory Unit on Ageing and Health, Vestfold Hospital Trust, Tønsberg, Norway; 3 Department of Geriatric Medicine, Oslo University Hospital, Oslo, Norway; 4 Research Centre for Age-related Functional Decline and Disease, Innlandet Hospital Trust, Brumunddal, Norway; 5 Norwegian Polar Institute, Tromsø, Norway; 6 Department of Health Science Gjøvik, Faculty of Medicine and Health Sciences, NTNU, Gjøvik, Norway; 7 Faculty of Medicine, University of Oslo, Oslo, Norway

**Keywords:** Ageing, Intrinsic capacity, Cognition, Physical capability, Disability, Frailty, Longevity, Quality of life

## Abstract

**Purpose:**

The Norwegian Survey of Health and Ageing (NORSE) was set up to provide internationally comparable data on ageing in Norway, which includes measured intrinsic capacity and cognitive function.

**Participants:**

NORSE is a population-based health examination study of seniors aged 60+ from the 1921–1958 birth cohorts in the former Norwegian county of Oppland, interviewed and examined during 2017–19 (*N* = 957, 16% response rate). NORSE is to some extent based on the SHARE-questionnaire (share-project.org), which includes work-related information, self-assessed and retrospective health, and expectations on longevity, quality of life, volunteering activities, consumption, and financial arrangements. In addition, several objective measures of intrinsic and cognitive capacity are included in NORSE.

**Findings to date:**

A shorter preferred life expectancy (PLE) was found to be associated with the prospects of a life with dementia and chronic pain. Motivation for retirement was found to be related to work-life experience and health. Social media was mostly used in the younger age groups and there was a tendency towards more use in the higher educational groups. NORSE incorporates questions on religion, and older women tend to have a higher degree of religiosity (proxied as self-assessed religiosity) than men in their 80s, but more similar (and lower levels) among those in their 60s.

**Future plans:**

NORSE participants have allowed their data to be linked to National registry data and midlife health examination studies and thereby provide a longitudinal design as well as information on disability status, socioeconomic status, household and marital status, support to/from children and parents, and pension status.

**Supplementary Information:**

The online version contains supplementary material available at 10.1186/s12889-021-12294-3.

## Strengths and limitations of this study

• A key strength of NORSE is the combination of objective measured data on health and functioning (e.g., grip strength, cognitive functioning) with economic and social information (e.g., retirement, intergenerational support).

• Another strength is the possibility to link data to a wide range of registries, and the overlap of the birth cohorts with earlier health examination data, of which participants have agreed to linkage, where a large share of the study cohort was extensively examined during midlife.

• Thus, NORSE makes it possible to investigate ageing longitudinally from midlife into older age.

• A weakness of NORSE is that it is regional and confined to only one Norwegian county, and that the response rate is only 16%. Still, data is representative for the former Oppland County when it comes to age and sex.

## Introduction

The Norwegian Survey of Health and Ageing (NORSE) was set up due to a lack of internationally comparable data on ageing in Norway, which includes measured intrinsic capacity and cognitive function. The World Health Organization (WHO) defined in 2015 healthy ageing as an “ongoing process of developing and maintaining the functional ability that enables well-being in older age”, and thereby shifting focus from diseases to functional ability, which is the interaction of the person’s intrinsic capacity and their environment [1]. Of key importance is collection of data on intrinsic capacity, and two such indicators – delayed word recall and grip strength, with comparable data from 36 countries were presented in the baseline report for Decade of healthy ageing [2]. NORSE adds Norwegian data on these important indicators.

Nationally representative surveys on health and ageing with harmonized tests and questions already cover more than half the world’s population, including HRS (for the United States), SHARE and ELSA (for more than 20 European countries), and SAGE (covering India, China, South Africa, Mexico, Ghana and Russia) [2, 3]. Norway lacks a health and ageing survey that is harmonized with these surveys - which includes  capacity tests and questions that is comparable with these surveys. These ageing surveys allow us to have comparable data in a comprehensive standardized fashion on intrinsic capacity, as well as physical health, frailty and disability status, mental health, chronic conditions, cognition, living arrangements, ability to take care of oneself, work and pensions, risky health behaviour, such as alcohol use and smoking, family relations, and economic situation. NORSE complements the The Norwegian Life Course, Ageing and Generation Study (NorLAG), which is based on self-reports and registry linkage, lacking objective measures of function and capacity [4]. NORSE also complements the large population based Tromsø study [5] and HUNT study [6], by the inclusion of questions specific to ageing, not included in these studies, such as expectations on longevity.

In Norway, administrative registers can be linked to the data by the unique personal identification number and provide information on disability, diseases, mortality, socioeconomic position, pension, marital status, support from children and parents, and more. Moreover, the longitudinal dimension in NORSE will follow from linking objective tests from several life-course stages, including extensive mid-life health examinations from the Norwegian Counties Study performed by The National Mass Radiography Service (35–49 year olds, both genders, up to three waves, tested 1976–88) [7], which is largely overlapping with our birth cohorts. NORSE is a collaborative effort between Department of Health Sciences Gjøvik NTNU, the Norwegian Institute of Public Health, the Norwegian National Advisory Unit on Ageing and Health, and Innlandet Hospital Trust.

NORSE is a health examination of seniors from the 1921–1958 birth cohorts in the former Norwegian county of Oppland, where 4% of the Norwegian population lives, and will accommodate the lack of data on birth cohorts which can be followed over the adult life cycle. Oppland was one of 19 Norwegian counties until January 1st 2020, when Oppland and Hedmark were merged into Innlandet county. Oppland county was situated north of Oslo, it had 189,545 inhabitants in 2019 (4% of the total Norwegian population), consisted of 26 municipalities, and Lillehammer as the administrative center [8]. The county was mostly rural, with farming and forestry as important contributors to the economy, and in recent years, tourism was also an important contributor. Immigrant population was 9.5% in 2019 (25.2% in Oslo), and the population was the second oldest county with 28% 60+ year olds in 2019 (17% in Oslo) [8]. Among the 60+ population of Oppland 0.6% emigrated during 2019 compared to 0.7% in Norway and 1.6% in Oslo [8].

We performed a pilot study in 2014, where we tested out the data collection strategy. Based on the positive feedback from this pilot the full-scale survey was conducted 2017–2019. The time lag between the pilot and the full survey was due to lack of funding, and logistics. Similar to the pilot, the full-scale survey included face-to-face-interviews to gather objective measurements on physical and cognitive performance, as well as anthropometric measures, and blood pressure. A questionnaire was used, which includes measurements on a range of health, social, economic, household, and demographic information. The questionnaire is compatible with other European, and non-European surveys, of ageing, and it was based on the harmonized SHARE version 5 (share-project.org) questionnaire. The questions include work-related information, self-assessed and retrospective health, and expectations on longevity, quality of life, volunteering activities, consumption, and financial arrangements. See appendix for list of variables included in both SHARE (version 5) and NORSE. Administrative registers will be linked to the data by the unique personal identification number and provide information on disability status, socioeconomic status, household and marital status, support to/from children and parents, and pension status. A key strength of NORSE is the combination of data on health and functioning with economic and social information (e.g., retirement, intergenerational support).

## Cohort description

The cohort includes a sample of participants aged 60 years and above living in the former county of Oppland, Norway [8].

### Sampling scheme

The Norwegian Tax Administration gave permission to draw a random sample from the National Population Register of *N* = 6000 participants aged 60 years and above residing in the former county Oppland. The data was collected over a period of 3 years, during the months February and March in 2017, 2018 and 2019, and in each of these years, an age stratified, random sample of *n* = 2000 was drawn without replacement. The total sample summed up to *N* = 5981 unique individuals, as 19 participants lacked a valid address and were removed. Each of the drawn individuals were assigned a unique NORSE ID-code, and the bridge linking this code with the personal identification number is stored at the Norwegian Institute of Public Health, apart from all data, and without access for the NORSE project group. The three age strata were 60–69, 70–79 and 80+ years, with equal numbers drawn from each age group, and thereby achieving oversampling of the older age groups (Table 1). Age on January 1st the 3 years was used.

**Table 1:** Study population and population in Oppland County per January 1st 2017, by sex, age and education (*N* = 957)

	NORSE Study population^a^		Oppland County, January 1st 2017	
	N	%	N	%
Total	957	100,0%	55,507	100,0%
Men	475	50,1%	24,184	47,0%
Women	473	49,9%	27,323	53,0%
Age groups				
60–69	386	40,7%	24,259	47,1%
70–79	381	40,2%	17,158	33,3%
80+	181	19,1%	10,090	19,6%
Education				
Compulsory	221	24,3%	17,943	35,1%
Secondary	312	34,4%	18,752	36,7%
Tertiary	375	41,3%	14,444	28,2%

^a^9 had missing information about age/sex and 40 for education. When linked to the Population registry information will be updated for these participants

### Recruitment strategy

To raise awareness of the study, local newspapers and radio were approached and they had coverage of the study the week before start of recruitment [9]. Eligible participants were mailed, in regular post, a four-page leaflet and invitation letter with detailed description of the study aims, the testing procedures, and how data would be handled after the data collection. The leaflet contained ethical clearances and consent procedure, as well as how participants later could withdraw their consent at any time. Those willing to participate could either send a mobile text message, or sign up using a pre-paid letter. Two weeks after the initial invitation letter were posted, a reminder was mailed to non-responders. A total of 957 out of 5981 invited participated in the interviews and health examinations (16%).

### Data collection

A pilot study was performed in 2014, where the data collection strategy was tested out. Both in the pilot study and in the full study, final-year nursing students at Department of Health Sciences Gjøvik, who were specifically trained for the data collection, contacted the participants and scheduled a physical meeting for interview and examination, either at home or in local healthcare clinics or offices. At the time of testing, the respondents signed an informed consent. The full study was performed over 3 years, with three cohorts of final year nursing students collecting the data; in 2017, 87 nursing students had 1–13 interviews each (median 4), in 2018, 105 nursing students had had 1–8 interviews (median 2), and in 2019, 110 students performed 1–6 interviews (median 3). Comparable interviews and data collections have been performed in other studies, and we had positive experiences from our pilot-study. In a US study of more than 5000 participants aged 71 years and older, the same physical performance battery (SPPB) we used in NORSE was used and no injuries resulted from the administration of the performance tests [10]. The same applied to the NORSE pilot and main study. During the interview, all data was written into a standardized protocol by the nursing student. The data were later scanned and cleaned and transferred into the statistical software SPSS. Further data cleaning and file preparation was done in SPSS and Stata. Finally, a harmonised data file, containing data from all 3 years was prepared.

### Sample size and response rates

Among the 5981 sampled 60+ year olds, 957 participated and the overall response rate was 16%, with higher response in the youngest age groups 60–69 years and 70–79 years (both 19%) compared with the older age group 80+ (9%). Number of respondents during the 3 years were 342 (17%) in 2017, 321 (16%) in 2018 and 294 (15%) in 2019, respectively. NORSE participants included birth cohorts (bc) born during 1921–58. The sample in 2017 covered bc 1921–56 with age range 60–95 years at study, while the sample drawn in 2018 covered bc 1921–57 with age at study 60–96 years, and finally in 2019 the bc was 1922–58 with age at study 60–96 years. Sampling during the 3 years was performed without replacement. Hence, the same person could be included only once. The NORSE sample is representative regarding age and sex, but response rate was higher among those with higher education (Table 1), and it is likely that we have a healthy selection bias.

## How often will they be followed up?

No additional data collection is planned in NORSE, but the participants have consented for linkage to a wide range of national registry data and to earlier health surveys in the former Oppland County. Of special interest is the earlier population-based health examination studies in Oppland County, which took place three decades prior to NORSE. Most of the NORSE study participants have also participated in the Oppland County study (see Table 2). Thus, it will be possible to construct a longitudinal study with two or more repeated measurements, where the same individuals are measured both in midlife and in older age. During 1976–78 all men and women aged 35 to 49 years, living in Oppland County were invited to a cardiovascular health survey [7]. The participants were re-invited to similar follow-up surveys in 1981–83 and 1986–88, in addition to refresher samples. In total, 28,068 persons 35–49 years old were invited and 25,851 participated (92%). These were born 1925–41 and largely overlapping the NORSE birth cohorts born 1921–58. Similar procedures and questionnaires were used in the 40-years old surveys (“40-årings undersøkelsene”) in 1991, and in 1997–98 in Oppland County, where 40–42 years old were invited in 1991 and 7820 in 1997–98 (*N* = 13,196). These were aged 60–70 years in 2017 [11]. Linkage has not yet been performed, but based on numbers in Table 2, we estimate that 58% of the NORSE participants participated in one or more of these previous health examination studies. Variables, which is on file at Norwegian Institute of Public Health, are measured height, weight, blood pressure, cholesterol level, triglycerides, blood glucose, and self-reports on time since last meal, history of heart disease and diabetes and/or symptoms, physical activity, smoking, alcohol use, work-life and working activity.

**Table 2:** Previous health examination studies in Oppland during 1970s – 990 s, which can be linked to NORSE

	County	Year	Invited (N)	Participated (%)**	Birth cohort	Age at survey	Age in 2017
Counties Study	Oppland	Wave I: 1976–8	*N* = 28,068	92%	1925–41	35–49	76–92
Counties Study	Oppland	Wave II: 1981–3	–	91%	1925–41	40–58	76–92
Counties Study	Oppland	Wave III: 1986–8	–	87%	1925–41	45–63	76–92
40-year surveys	Oppland	1991	N = 13,196	73%	1947–51	40–42	66–70
40-year surveys	Oppland	1993–4	*N* = 7532	73%	1952–54	40–42	63–65
40-year surveys	Oppland	1997–8	*N* = 7820	68%	1955–57	40–42	60–62

Source: https://www.fhi.no/div/helseundersokelser/landsomfattende-helseundersokelser-lhu/helseundersokelser/fylkesundersokelsene-i-finnmark-sog/

** Estimated from page 66–67 from this report https://www.fhi.no/globalassets/dokumenterfiler/studier/helseundersokelsene/oversikt-over-fylker-og-arskull-40-aringsundersokelser.pdf

Based on numbers in Table 2 (92% of the birth cohort 1925–41, 73% of the 1947–54 cohorts and 68% of the 1955–57 cohorts participated) we estimate that 553 (58%) NORSE participants will have participated in one or more of these previous health examination studies in the former county of Oppland

## What has been measured?

The full-scale NORSE survey included face-to-face-interviews and health examination to gather validated objective measurements on physical and cognitive capacity, as well as anthropometric measures (height, weight, and waist circumference), and blood pressure (see Table 3). In addition, self-reported validated questionnaire data includes measurements on a range of health, social, economic, household, and demographic information. The questionnaire is compatible with other European (and non-European surveys) of ageing, and it is based on the harmonized SHARE (share-project.org) questionnaire. The questions include rich work- and pension related information, education (own and spousal), marital status, information on siblings and children/grandchildren, volunteering activities, travel distance to relatives, social contact with friends and relatives, social media use, financial arrangements, risk factors such as inactivity, smoking and alcohol use, self-assessed and retrospective physical and mental health, ADL, vision, hearing, diseases, medicine use, quality of life, loneliness, health services use. In addition, NORSE includes unique questions on expectations on longevity and preferred life expectancy (Table 3) [12]. NORSE has information about whether a proxy was present and helped to answer the questions or interfered the interview. Furthermore, the interviewers provided information on whether the respondent understood the questions and whether there was fatigue during the interview. Danish and Swedish translations of the SHARE-questionnaire exist, and these were used with the original English version to make a Norwegian translation. Full population data from the former county of Oppland for year 2017 by age, sex and education provided by Statistics Norway was used to create population weights, which can be applied to control for selection bias.

**Table 3:** Overview over measures in NORSE

Socioeconomic status and demography:	Housing, number of stairs at main entrance, education, income, marital status, spouse education, age and birth year of parents, residency of parents, employment/working situation, type of employment, age at retirement, reason for retirement, feelings after retirement, job satisfaction.
Social contact and assistance:	Social contact with parents, provision of help to/from parents, siblings, children, social contact with children/grandchildren, provision of help to/from children/grandchildren, social contact with friends.
Health and physical function (self-reported):	Self-reported health status, parents´ health, longstanding limiting illness which affects functions in daily life, diseases, medications, symptoms, vision, hearing, hearing aid, pADL, iADL, depression (EURO-D scale) [20], anxiety (Generalized Anxiety Disorder Screener, GAD-7) [21], sleep quality, vitality, subjective age, subjective life expectancy, preferred life length, hypothetical conditions affecting preferred life length.
Anthropometrics (measured):	Systolic and diastolic blood pressure x 2, pulse, height, weight, waist circumference, arm circumference
Intrinsic capacity (measured):	Grip strength (JAMAR dynamometer), Short physical performance battery (SPPB, the official Norwegian version [22]) (standing balance, walking speed, chair rise test), one-leg standing balance (eyes open/closed) [23, 24], Montreal Cognitive Assessment (MoCA) [25], 10-word memory test (immediate and delayed recall), Cognitive Function Screening Instrument (MCFSI) (KFI) self-reported [26].
Health related factors:	Smoking, snus, alcohol use, physical activity, loneliness, volunteering, leisure activities.
Health services use:	Use of general practitioner, medical specialist by type, dentist, nursing home, home based care.
Other:	Religiosity

## Findings to date

NORSE data has only recently been available for research and therefore output is limited, and publications are restricted to one peer reviewed research paper in the journal *Age and Ageing*, a master thesis, and three conference abstracts. Preferred life expectancy (PLE) was found to be associated with hypothetical adverse life scenarios among Norwegians aged 60 + [12]. Especially the prospects of a life with dementia and chronic pain was associated with shorter PLE. Furthermore, in preliminary work on health and function after retirement, presented at the Nordic Congress on Gerontology 2021 (NKG25), it was reported that those who were motivated to retire due to the good Norwegian public pension schemes and to enjoy life had a better work-life experience and also better self-reported health after retirement than those who retired due to poor health or being tired of work [13]. Another, presentation at NKG25 investigated social media use in the elderly, and reported more use in the younger age groups and a tendency towards more use in the higher educational groups [14, 15]. Quantification of the feasibility using nursing students for data collection in the NORSE pilot study was presented at the Nordic Congress on Gerontology 2014 (NKG22) in Gothenburg, Sweden [16].

We include two graphs, exemplifying findings from NORSE on one measure of physical functioning (pace of walking) and one social variable (self-assessed religiosity).

**Fig. 1:**
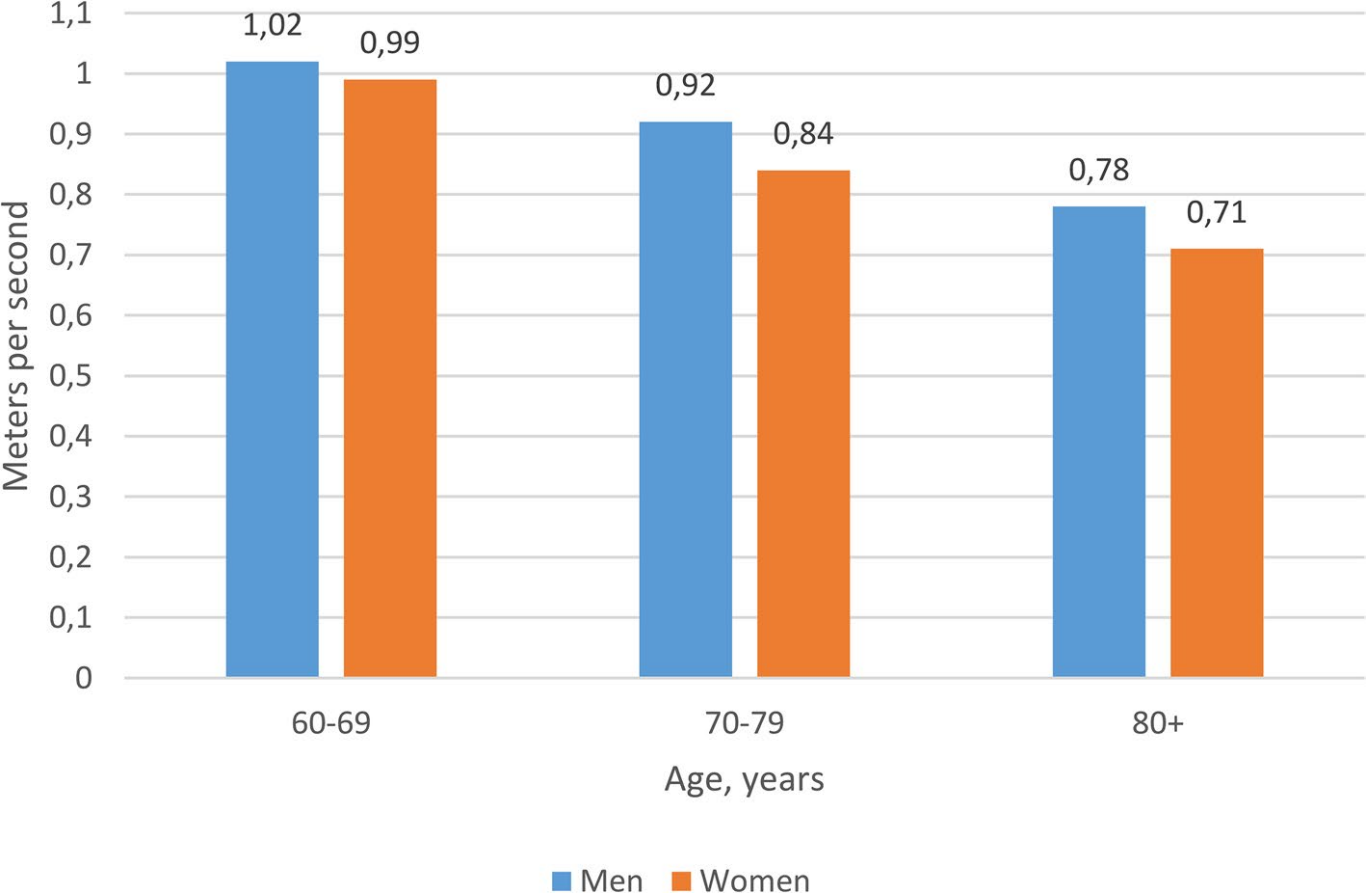
Mean walking speed (meters per second) by age and sex. Number of participants: 298. Age trend: *p* < 0.01 for both men and women

Pace of walking: Walking speed among older individuals may predict the risk of several health outcomes, including all-cause mortality [17]. Evidence from NORSE, see Fig. 1, reveals a lower walking speed among those in their 70s and 80s compared to those in their 60s; and that the age-related variation in walking speed is stronger for women.

**Fig. 2:**
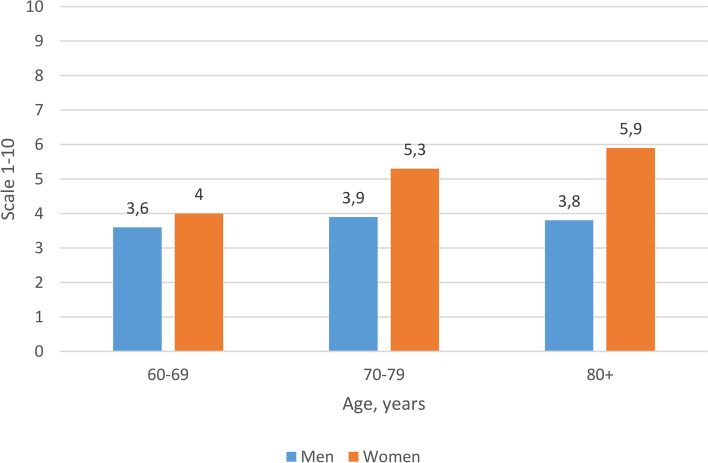
How religious would you describe yourself on a scale of 0–10? (of which 10 are most religious). Mean values by age and sex. Number of participants: 297. Age trend: women *p* < 0.01; men *p* = 0.59

Belief in God: Religious beliefs have been found to be associated with health and demographic outcomes [18, 19]. NORSE incorporates questions on religion – Fig. 2 shows that older women tend to have a higher degree of religiosity (proxied as self-assessed religiosity) than men in their 80s, but more similar (and lower levels) among those in their 60s.

## Strengths and limitations

A key strength of NORSE is the combination of objective measured data on health and functioning with economic and social information (e.g., retirement, intergenerational support). Another strength is the possibility to link data to a wide range of registries, and the overlap of the birth cohorts with earlier health examination data, of which participants have agreed to linkage, where a large share of the study cohort was extensively examined during midlife. Thus, NORSE makes it possible to investigate ageing longitudinally from midlife into older age. A weakness of NORSE is low response rate. Still, data is representative when it comes to age and sex.

## Supplementary Information

**Additional file 1.**

## Data Availability

To get access to NORSE data for medical research, each project will need a specific clearance from the Regional Ethics Committee (REC) and the NORSE project group. The REC application and REC approval is sent to the NORSE study along with an application. More information is found at the NORSE web site at the Norwegian Institute of Public Health: https://www.fhi.no/studier/norse-studien/.
