# Supplementary material for: Cohort profile: Norwegian survey of health and ageing (NORSE)
Source: BMC Public Health. 2021 Dec 8;21:2229. doi: 10.1186/s12889-021-12294-3 (PMC8653560; doi:10.1186/s12889-021-12294-3)
Supplement: Supplementary file 1 — Additional file 1. [file 12889_2021_12294_MOESM1_ESM.docx]

# Appendix

*Appendix table: Topics covered in SHARE (wave 5) which are also included in NORSE*

| **SOCIOECONOMIC STATUS AND HOUSING** |
| --- |
| Which type of building does the household live in? |
| How many steps had to be climbed (up or down) to get to the main entrance of the household's flat? |
| What is the highest school leaving certificate or school degree that you have obtained? |
| **INCOME, EMPLOYMENT AND PENSIONS**  (current/previous work, sickness, reasons for retirement) |
| Thinking of your household's total monthly income, would you say that your household is able to make ends meet ... |
| Current situation (retired, employed, unemployed, sick, disabled, homemaker, other) |
| Have you ever done any paid work? |
| Type of job, work hours, and position at the work place (several items identical to SHARE) |
| Job satisfaction, demands and autonomy (several items identical to SHARE) |
| Thinking about your present would you like to retire as early as you can from this job? |
| Are you afraid that your health will limit your ability to work in this job before regular retirement? |
| Since you stopped working, has retirement mainly been a relief or a concern for you? |
| In your last job, were there opportunities to work, either full time or part-time, after the official retirement age? |
| **FAMILY** |
| What is your marital status? |
| What is the highest school certificate degree that [your/your/your/your/your/your] or [husband/wife/husband/wife/husband/wife] has obtained? |
| Is [your/your] [natural/natural] [mother/father] still alive? |
| Where does parent live? |
| Grandchildren, number of.. |
| How would you describe the health of [your/your] [mother/father]? |
| Have you ever had any siblings? |
| Were you the oldest child, the youngest child, or somewhere in-between? |
| How many brothers do you have that are still alive? |
| How many sisters do you have that are still alive? |
| How many children do you have that are still alive? Please count all natural children, fostered, adopted and stepchildren[, including those of/, including those of/, including those of/, including those of/{empty}/{empty}] [your husband/your wife/your partner/your partner/{empty}/{empty}]. |
| **SOCIAL CONTACT AND ASSISTANCE** |
| During the past twelve months, how often did you [or your/or your/or your/or your//  [husband/wife/partner/partner/ have contact with [{child}], either personally, by phone or mail? |
| During the past twelve months, how often did you have contact with [your/your] [mother/father], either personally, by phone or mail? |
| **HEALTH AND PHYSICAL FUNCTION** |
| Would you say your health is ... |
| Some people suffer from chronic or long-term health problems. By long-term we mean it has troubled you over a period of time or is likely to affect you over a period of time. Do you have any long-term health problems, illness, disability or infirmity? |
| For the past six months at least, to what extent have you been limited because of a health problem in activities people usually do? |
| Has a doctor ever told you that you had any of the conditions (on this card)? |
| For the past six months at least, have you been bothered by any of the health conditions (on this card)? |
| Current drug use (at least one week) |
| Is your eyesight [using glasses or contact lenses as usual/]... |
| Do you usually wear glasses or contact lenses? |
| How good is your eyesight for seeing things at a distance, like recognising a friend across the street [using glasses or contact lenses as usual/]? Would you say it is ... |
| How good is your eyesight for seeing things up close, like reading ordinary newspaper print [using glasses or contact lenses as usual/]? Would you say it is ... |
| Are you usually wearing a hearing aid? |
| Is your hearing [using a hearing aid as usual/...]… |
| Do you find it difficult to follow a conversation if there is background noise, such as a TV, a radio or children playing [using a hearing aid as usual/]? |
| Can you hear clearly what is said in a conversation with several people? |
| Can you hear clearly what is said in a conversation with one person [using a hearing aid as usual/]? |
| Do you use dentures? |
| [Using your dentures,/] [can you/Can you] bite and chew on hard foods such as a firm apple without difficulty? |
| **DISABILITIES AND ACTIVITIES OF DAILY LIVING** |
| \| Please tell me whether you have any difficulty doing … \| \| --- \| \| Walking 100 metres \| \| Sitting for about two hours \| \| Getting up from a chair after sitting for long periods \| \| Climbing several flights of stairs without resting \| \| Climbing one flight of stairs without resting \| \| Stooping, kneeling, or crouching \| \| Reaching or extending your arms above shoulder level \| \| Pulling or pushing large objects like a living room chair \| \| Lifting or carrying weights over 10 pounds/5 kilos, like a heavy bag of groceries \| \| Picking up a small coin from a table \| |
| \| Here are a few more everyday activities. \| \| --- \| \| Please tell me if you have any difficulty with these because of a physical, mental, emotional or memory problem… \| \| Dressing, including putting on shoes and socks \| \| Walking across a room \| \| Bathing or showering \| \| Eating, such as cutting up your food \| \| Getting in or out of bed \| \| Using the toilet, including getting up or down \| \| Using a map to figure out how to get around in a strange place \| \| Preparing a hot meal \| \| Shopping for groceries \| \| Making telephone calls \| \| Taking medications \| \| Doing work around the house or garden \| \| Managing money, such as paying bills and keeping track of expenses \| |
| Thinking about the activities that you have problems with, does anyone ever help you with these activities? |
| Would you say that the help you receive meets your needs? |
| **ANTHROPOMETRICS** |
| Approximately how much do you weigh? (kg) |
| How tall are you? (cm) |
| **INTRINSIC CAPACITY (measured)** |
| Hand grip strength |
| Walking speed |
| TEN WORDS LIST LEARNING DELAYED RECALL |
| **HEALTH RELATED FACTORS** |
| Smoking (several items) |
| Alcohol (several items) |
| Physical activity |
| **MENTAL HEALTH** |
| Mental Health complete EURO-D |
| LONELINESS (ONE GLOBAL ITEM) i senere SHARE ELLER HER? |
| **HEALTH SERVICES USE** |
| Contact with general practitioner (several items) |
| Contact with specialist (several items) |
| Contact with specialist (two items) |
| During the last twelve months, have you been in a nursing home overnight? |
| Received home care in own home? |
